# Enhancing Febuxostat Solubility Through Cocrystal Formation: Role of Substrate Selection and Amide Coformers

**DOI:** 10.3390/ijms26073004

**Published:** 2025-03-26

**Authors:** Edyta Pindelska, Anita Sarna, Maciej Duszczyk, Anna Zep, Izabela D. Madura

**Affiliations:** 1 Faculty of Pharmacy, Medical University of Warsaw, Banacha 1, 02-093 Warsaw, Poland; 2 Scientific Circle “Spektrum”, Faculty of Pharmacy, Medical University of Warsaw, Banacha 1, 02-093 Warsaw, Poland; anita.sarna@wp.pl (A.S.); maciej.duszczyk@onet.pl (M.D.); 3 Analytical Research Section, Pharmaceutical Analysis Laboratory, Łukasiewicz Research Network, Industrial Chemistry Institute, Rydygiera 8, 01-793 Warsaw, Poland; anna.zep@ichp.lukasiewicz.gov.pl; 4 Faculty of Chemistry, Warsaw University of Technology, Noakowskiego 3, 00-664 Warsaw, Poland; izabela.madura@pw.edu.pl

**Keywords:** febuxostat, cocrystals, syntheses, solubility, dissolution rate, grinding, polymorphism

## Abstract

Solubility plays a crucial role in drug bioavailability and therapeutic efficacy. Febuxostat (FEB), a BCS Class II drug used to treat hyperuricemia and gout, has low solubility, limiting its effectiveness. Cocrystallization offers a strategy to enhance solubility without modifying the drug’s chemical structure. While FEB exhibits multiple polymorphic forms, no prior studies have explored cocrystal formation from its commercially available hemihydrate. This study examines whether FEB’s initial form—hemihydrate or anhydrous—affects cocrystal formation. We investigated cocrystals with aromatic amides (nicotinamide, isonicotinamide, and picolinamide) and explored new FEB cocrystals with aliphatic amides (diacetamide, malonamide, and D,L-lactamide) to assess solubility enhancement. Our results show that anhydrous FEB cocrystals reliably form with both aromatic and aliphatic amides, regardless of the starting material. However, the aliphatic coformers lead to thermally unstable cocrystals. Nevertheless, the new cocrystals significantly improved FEB’s solubility, with FEBH-LAC (13.9 mg/L) being the most soluble, but thermally unstable. FEBH-DIA showed the best balance, with 12.2 mg/L solubility and the fastest dissolution rate. These findings highlight cocrystallization with aliphatic amides as a promising approach for enhancing FEB’s solubility and therapeutic potential; however, they may pose problems with stability and reproducibility.

## 1. Introduction

Modern pharmacotherapy is based mainly on two important components: the safety of substances administered to the patient and the effectiveness of treatment. Solubility—a physicochemical feature of the active pharmaceutical ingredient (API)—has a direct impact on these properties. In oral drug delivery systems, gastrointestinal absorption significantly depends on the solubility and dissolution rate of drug molecules [1]. Upon oral administration, a drug traverses the digestive system, requiring dissolution for absorption into the bloodstream. Once absorbed, the active compound partially binds to plasma proteins, while the unbound fraction interacts with target receptors to elicit its therapeutic effect [2]. Thus, the solubility of the active substance is a critical determinant of its bioavailability and pharmacological efficacy. Importantly, increasing the drug’s solubility decreases the required dose to achieve the therapeutic effect, which in turn minimizes adverse effects and enhances the active substance’s safety profile. Currently, around 90% of new chemical entities and 40% of marketed drugs fall into Biopharmaceutical Classification System (BCS) classes II and IV, characterized by poor water solubility and low bioavailability [3]. To address this challenge, the pharmaceutical industry is actively exploring methods to enhance drug solubility, with cocrystallization emerging as a promising approach [4,5,6]. Its key advantage lies in the ability to modify cocrystal properties using pharmaceutically acceptable co-formulants without altering the chemical structure of API [7]. Beyond improving solubility [8], cocrystallization can also influence hygroscopicity, stability [9], and production parameters such as flow, thickening, and processability [5]. This versatility makes it a valuable tool for optimizing various drug properties when designing new solid forms. Pharmaceutical cocrystals are defined as crystalline single-phase materials composed of two or more different molecular and/or ionic compounds, typically in a specific stoichiometry ratio that are neither solvates nor simple salts [10]. According to the FDA, cocrystals represent a unique class of solvates in which the second component is non-volatile [11].

**Figure 1:**
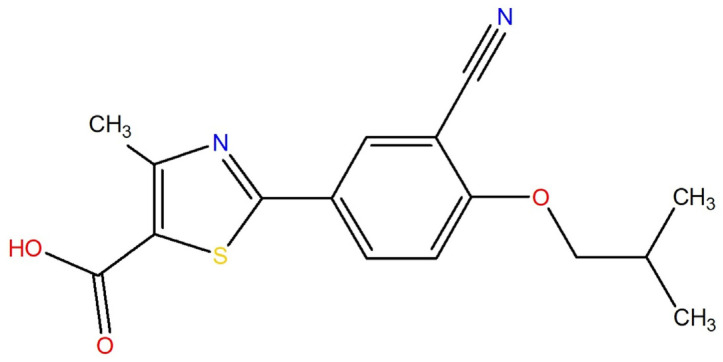
Chemical structure of FEB.

Febuxostat (2-(3-cyano-4-isobutoxyphenyl)-4-methyl-1,3-thiazol-5-carboxylic acid, FEB, Figure 1) is a thiazolocarboxylic acid derivative belonging to the non-purine xanthine oxidase inhibitors, used to treat chronic hyperuricaemia and gout [12]. Discovered by Teijin in 1996, it was approved in the EU as Adenuric in 2008 and by the FDA as Uloric in 2009. FEB exhibits numerous polymorphic forms, with various patents documenting different crystalline structures. In US patents, these forms are labeled F1–F14 [13], while European patents use designations A, B, C, D, and G [14], and WO documents describe forms H, I, and J [15]. Additionally, Chinese patent publication discloses crystalline form Q [16]. The inconsistent nomenclature creates challenges in accurately identifying and comparing these forms. As a Biopharmaceutical Classification System (BCS) class II drug, FEB has low solubility but high permeability [17,18], and each polymorphic form may differ in solubility, stability, or dissolution rate. Moreover, each of these forms can serve as a starting material for developing new crystalline FEB forms with enhanced properties. Beyond cocrystals, salts, and solvates, multicomponent systems—such as hydrate/solvates of salts and cocrystals or ionic cocrystals, including their solvated forms—may offer additional advantages [19]. Cocrystal hydrates, in particular, present a promising strategy for improving stability under high-humidity conditions [20]. Their synthesis can be, for example, achieved using API crystalline hydrates as substrates. To the best of our knowledge, there are no reports on syntheses of FEB multicomponent crystals starting from commercially available febuxostat hemihydrate, FEBH.

This study aims to determine whether the choice between FEBH and anhydrous forms influences the formation of cocrystals. To investigate this, we examined cocrystals with nicotinamide, isonicotinamide, and picolinamide, for which either a single crystal data [21,22] or powder diffraction data are available [23]. However, developing novel FEB modifications is essential to enhance its solubility and potentially reduce the required drug dose, especially in light of the FDA’s 2019 warning [24] about an increased risk of heart-related death associated with the Uloric drug compared with Allopurinol (other medicine for gout). Therefore, we also aimed to design and obtain new FEB cocrystals with improved solubility.

## 2. Results and Discussion

### 2.1. Choice of Substrate Crystalline Form

Using crystal engineering tools to design new solid forms [25] of API, one has to begin with the inspection of crystals of starting materials. As mentioned above, FEB is highly polymorphic; however, only three polymorphic forms have been structurally characterized and deposited in the Cambridge Structural Database (CSD) [26] so far, and labeled there as Q, A, and H1. Historically, the first fully described form was a monoclinic (Q) one obtained from solution crystallization from acetonitrile [21]. The second was a metastable triclinic H1 polymorph obtained by slow evaporation from ethyl acetate–toluene solution [27]. Very recently, the orthorhombic polymorph A, being a commercially available form, has been described [28]. Finally, in 2025, a hemihydrate structure, also commercially available (CSD refcode WOWSOU), in the literature sometimes described as form G was elucidated [29]. The latter paper compares form A and FEBH, indicating that, upon heating to ~200 °C, both forms undergo phase transition to form F10, labeled in the US patents, prior to melting above 210 °C.

**Figure 2:**
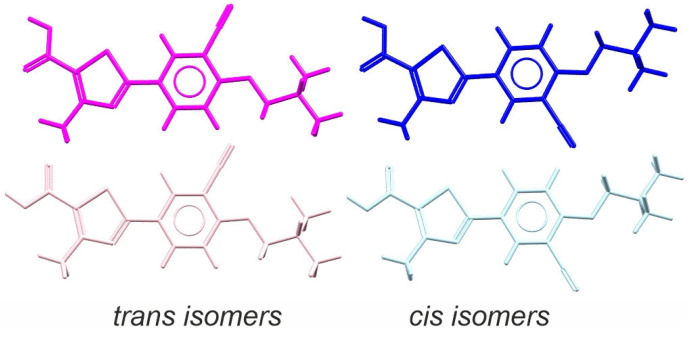
The most stable conformers of FEB. Calculations performed with the aid of Conformer Generator tool in CSD-Mercury program.

The crystal structure comparison revealed that FEB molecules can adopt different conformations in the solid state, influencing hydrogen bond patterns. Using the Conformer Generator tool in CSD-Mercury [30], we identified ten of the most stable conformations, four of which (Figure 2) have the potential to form distinct hydrogen-bonded synthons with coformers or solvent molecules. According to the nomenclature by Ilaveni et al. [29], FEB polymorphs exhibit both cis and trans conformers: form Q contains only the cis conformer, while forms A and H1 contain both cis and trans. In the FEBH, both crystallographically independent molecules adopt the cis conformation.

Notably, all structurally characterized FEB cocrystals were derived from polymorph A, with most adopting the trans conformation, as seen in cocrystals with acetamide (HIQQEF) [21], nicotinamide (HIQQIJ) [21], isonicotinamide (OYADAV) [22], urea (HIQQOP) [21], and 4,4′-bipyridine (QEDNUM) [31]. In contrast, cis conformers are present in cocrystals with 4-aminobenzoic acid (HIQQUV) [21] and piroxicam (RUNSUR) [32]. Additionally, all known solvates of FEB adopt the trans conformation, including those with ethanol (BUVBEC) [33], methanol (UREQOY) [34], acetic acid (XULRUT) [35], and pyridine (PUHGUV) [36].

The flexibility of the FEB molecule in forming different hydrogen synthons is further supported by experimental evidence of polymorph stability. When an equimolar mixture of form A and form G (hemihydrate) was stirred in acetonitrile for 24 h, complete conversion to form Q was observed [29]. These polymorphs exhibit distinct hydrogen-bonding arrangements: form A stabilizes through carboxylic acid dimers, while in form Q, the nitrile group acts as a hydrogen bond acceptor. In contrast, the FEBH features two types of synthons—carboxylic acid dimers and a tetrameric network involving two water molecules and two different conformers—making it the most energetically stable form [29].

**Figure 3:**
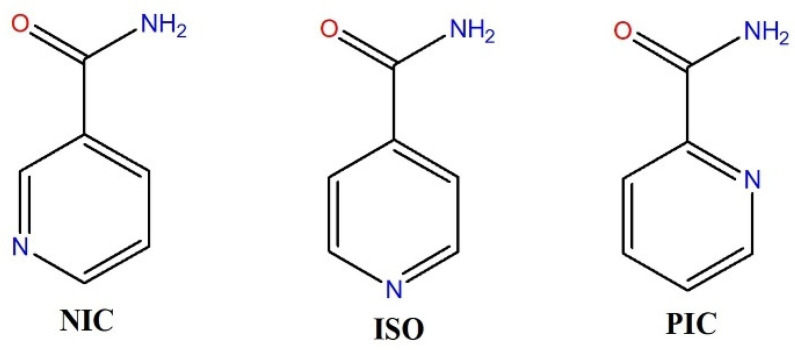
Isomers of pyridine–carboxamide used as coformers in this study: nicotinamide (NIC), isonicotinamide (ISO), and picolinamide (PIC).

Given these structural differences, we aimed to investigate whether using FEBH instead of polymorph A as a substrate could lead to the formation of new crystal forms. To explore this, we selected a series of isomeric coformers: nicotinamide (NIC), isonicotinamide (ISO), and picolinamide (PIC) (Figure 3)—although the crystal structure of the latter remains unknown.

The FEB-NIC, FEB-ISO, and FEB-PIC cocrystals (Figure S1) were obtained following previously described methods [21,22], with the modification of using FEBH as the starting material. The powder X-ray diffraction patterns of FEB-NIC and FEB-ISO fully match those calculated from their respective single-crystal structures, while the pattern for FEB-PIC closely resembles the one reported by Jagia et al. [23]. This suggests that the presence of water in the starting material does not impact cocrystal formation when the coformers contain two hydrogen bond donors and two hydrogen bond acceptors. In FEB-NIC, nicotinamide forms dimers that serve as hydrogen bond donors to the CN group and as acceptors for the hydroxyl group of febuxostat molecule. In FEB-ISO, the amide group forms a catemeric motif, linking to FEB through both the hydroxyl and cyclic nitrogen atoms. Notably, in both cocrystals, amide–amide interactions are present.

### 2.2. Rationale for Designing New Multicomponent Forms with Aliphatic Amides

A scrutinized literature search on FEB cocrystals reveals that the best equilibrium solubility in water is observed for those with L-arginine (570.8 mg/L) [22] and 3-aminopyridine (268.9 mg/L) [30]. In phosphate buffer (pH = 6.8), cocrystals with isonicotinamide (45.09 mg/L) and picolinamide (34.91 mg/L) show notable solubility [23]. Another study found that the FEB-acetamide cocrystal dissolves 52.5 times faster than FEB, but lacks stability, while the cocrystal with nicotinamide is stable and dissolves 36.6 times faster than FEB [21]. Although these dissolution tests were conducted in 60% ethanol—an imperfect medium for pharmaceutical cocrystals—they highlight solubility trends and enable comparisons.

**Figure 4:**
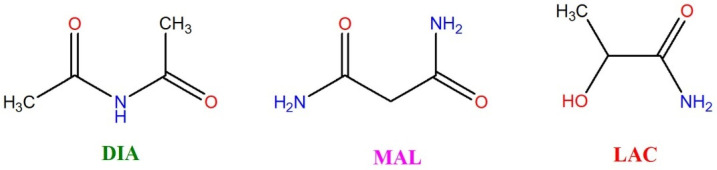
Coformers used in this study: diacetamide (DIA), malonamide (MAL), and D,L-lactamide (LAC).

These findings suggest that suitable FEB coformers include not only aromatic amides and amines, but also aliphatic amides. Based on this, we selected diacetamide (DIA), malonamide (MAL), and D,L-lactamide (LAC) for our studies. These basic molecules, with multiple hydrogen bonding sites (Figure 4), are promising candidates for cocrystal formation. Notably, all selected coformers are on the approved list for pharmaceutical salts and cocrystals [37].

### 2.3. Novel Cocrystals Synthesis and Characterization

Three novel cocrystals with DIA, MAL, and LAC were prepared from a FEBH by the liquid-assisted grinding method (LAG), with a drop of acetonitrile. The formation of the new phases was confirmed by infrared spectroscopy (FT-IR), powder X-ray diffraction (PXRD), and meting point comparison. The latter data are gathered in Table 1 and show that, in all cases, the cocrystal melts in the range between the melting point of FEBH and coformer.

The FTIR spectrum of the starting FEBH shows characteristic peaks at 2962, 2227, 1702, 1682, and 1511 cm^−1^ (Figures S2–S4, Table 2). They correspond to the single bond stretching of the O-H in the carboxylic group, C=N bond stretching of the thiazole ring, C=O bond stretching of the carboxylic group, and C≡N bond stretching, respectively. The carboxylic acid signal appears as a doublet signal at 1681 cm^−1^ (for the acid–acid dimer) and 1702 cm^−1^ (for the acid–water tetrameric motif) [29]. After the formation of the cocrystals, the band corresponding to the acid–acid dimer interaction shifted to lower frequencies in the cases of FEBH–DIA and FEBH–LAC to 1667 cm^−1^ and 1645 cm^−1^, respectively. However, in the case of FEBH–MAL, this band shifted toward higher ones, i.e., 1745 cm^−1^. This undoubtedly indicates the formation of a new pattern of hydrogen bonds between FEB molecules and the coformers.

The nitrile functional group also showed spectral shifts influenced by synthon variations in the analyzed crystal forms. The stretching frequency of the nitrile group that participates in C−H⋯N interaction in FEBH appears at 2227 cm^−1^ in the FTIR spectrum, when it is involved in nitrile–water interaction. After the formation of cocrystals, this band shifts by 12 cm^−1^ toward higher values to 2239 cm^−1^.

In the FTIR spectrum, the carboxylic acid dimers display very broad intense O−H stretching absorption in the 3300–2500 cm^−1^ region. These O−H stretching bands were usually centered near 3000 cm^−1^ and overlapped with C−H stretching vibrations [38]. Because FEB is a hemihydrate, it displays additional O−H stretching vibrations of water molecules at 2962 cm^−1^, 3459 cm^−1^, and 3534 cm^−1^. In the case of cocrystals, these bands overlap the N-H stretching vibrations of NH_2_ groups of the MAL and LAC coformers and NH of the DIA. In all cases, the N–H stretching bands are shifted due to the formation of acid···amide dimers.

**Figure 5:**
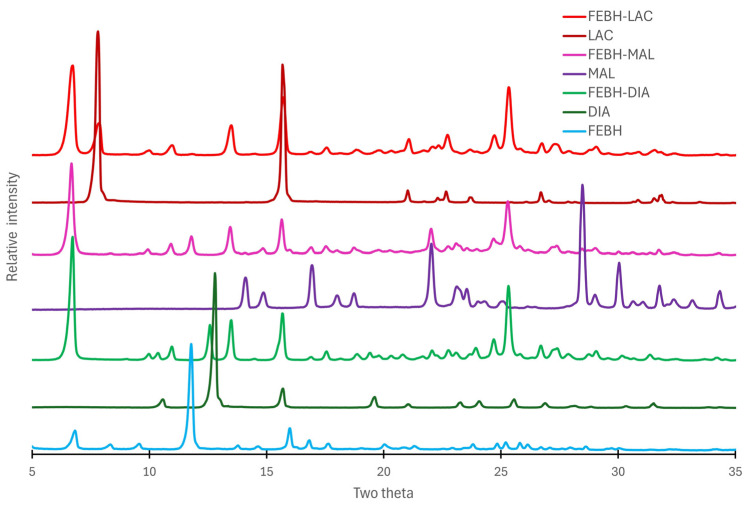
Powder X-ray diffraction patterns of FEBH, coformers, and cocrystals.

To univocally confirm the formation of cocrystals, we performed PXRD experiments. The PXRD patterns of FEBH and novel cocrystals FEBH-DIA, FEBH-MAL, and FEBH-LAC are depicted in Figure 5. The reflections in the FEBH diffractogram match those of the simulated-form single crystal ones and are fully consistent with the one described before [30]. Analysis of the positions of the diffraction reflections in the obtained samples after LAG revealed differences compared with the substrates’ diffractograms, and thus confirming the formation of the new crystalline phases. However, the positions of some reflections of the substrates are also present in the new phases. This might be a coincidence, or the stoichiometry of obtained cocrystals is different than assumed 1:1. Knowing that the patterns might be influenced by the presence of two crystalline solids, we also performed experiments using 1:1 physical mixtures, carefully avoiding any kind of grinding, even though we checked that the neat grinding synthesis does not lead to cocrystals and the patterns are identical to those of the physical mixture (Figures S5–S7).

### 2.4. Solubility, Dissolution Rate and Stability Studies

In the case of new pharmaceutical cocrystals, it is very important to determine their equilibrium solubility (thermodynamic property) and dissolution rate (kinetic parameter), because it is of great importance during the development of a new drug. Equilibrium solubility experiments for FEBH and its cocrystals were performed in water medium at 37 ± 0.5 °C. The results indicate that all three cocrystals improved the solubility of FEB. The equilibrium solubility values of FEBH, FEBH-DIA, FEBH-MAL, and FEBH-LAC cocrystals in aqueous medium were 7.6 mg/L, 12.2 mg/L, 12.8 mg/L, and 13.9 mg/L, respectively.

**Figure 6:**
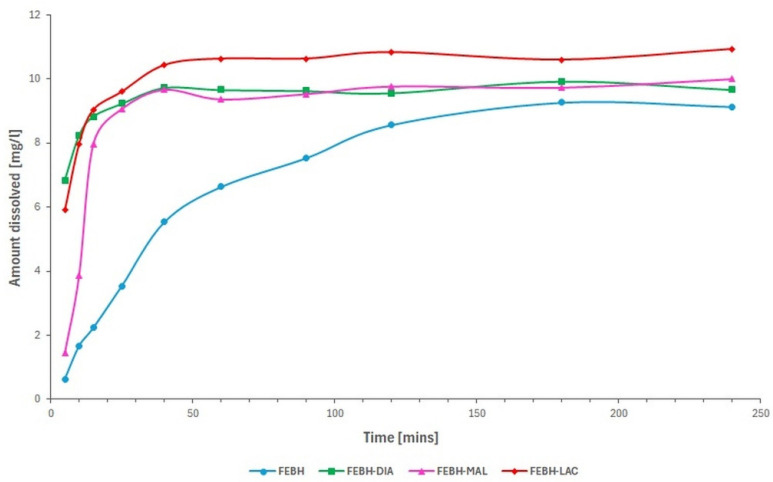
The dissolution rates of the FEBH–DIA cocrystal, FEBH–MAL cocrystal, FEBH–LAC cocrystal, and the FEBH pure drug in water. Note that the initial dissolution rate of the FEBH–DIA and FEBH–LAC cocrystals is significantly faster than that of the FEBH–MAL cocrystal and the parent FEBH.

The dissolution rate has a major impact on the performance and bioavailability of poorly water-soluble APIs [39]. In this study, powder dissolution experiments were conducted (Figure 6). Within 5 min, 6.9 mg/L FEB–DIA and 5.8 mg/L FEB–LAC dissolved, compared with only 0.6 mg/L for FEBH and 1.5 mg/L for FEM–MAL. Nearly peak concentrations were observed within 240 min for all samples. After 4 h of dissolution, the highest concentration was recorded for FEB–LAC (10.93 mg/L), followed by FEB–MAL (10.0 mg/L), FEB–DIA (9.67 mg/L), and FEB (9.13 mg/L).

The results of the PXRD analysis of the undissolved residue at the end of the dissolution experiment matched those of the initial cocrystals FEBH-DIA and FEBH-LAC, thus confirming the physical-form stability of the cocrystal in a dissolution slurry medium of water for 4 h (Figures S5 and S7). The FEBH-MAL cocrystal resulting from this experiment was a physical mixture (Figure S4), leading us to investigate the thermal stability of the cocrystals we studied.

In order to perform the differential scanning calorimetry (DSC) and thermogravimetric analysis (TGA) experiments, further samples of the cocrystals under investigation were prepared. In the case of FEBH-DIA and FEBH-LAC, the cocrystallization was reproducible, but in the case of FEBH-MAL, the cocrystal disappeared. Subsequent attempts to grind and recover the cocrystal were unsuccessful, and after performing PXRD. we observed a physical mixture. Despite these numerous attempts, this cocrystal could not be obtained.

Firstly, we confirmed the behavior of FEBH in DSC studies, with the data in agreement with the study by Ilaveni et al. [29]. We then carried out the DSC and TGA experiments on two of our promising cocrystals, FEBH-DIA and FEBH-LAC.

The DSC curve of the FEBH–DIA cocrystal (Figure S8) exhibited an early endothermic event with T_onset_ at 73.5 °C (T_peak_ = 75.9 °C; H_f_ = 26.6 J/g) and a second broad endothermic thermal effect with T_onset_ at 177.5 °C (T_peak_ = 189.9 °C; H_f_ = 94.9 J/g).

The DSC data of the FEBH–LAC cocrystal (Figure S9) also showed an early endothermic event with T_onset_ at 74.5 °C (T_peak_ = 76.8 °C; Hf = 42.3 J/g). As a result of further heating, a second endothermic transition with T_onset_ at 138.3 °C (T_peak_ = 166.4 °C; H_f_ = 65.8 J/g) followed by a third endothermic event with T_onset_ at 176.1 °C (T_peak_ = 179.4 °C; H_f_ = 2.8 J/g) were observed. In this case, we observes the transformation of one polymorphic form into a more thermodynamically stable one.

In order to investigate the nature of all endothermic thermal effects which have been observed in both DSC curves of the studied cocrystals (FEBH–DIA and FEBH–LAC) TG analysis was performed. The mass loss in the temperature range of 30–90 °C observed for both samples (Figures S11 and S12) did not exceed 1.0% (0.08% for FEBH–LAC and 0.56% for FEBH–DIA), suggesting that no dehydration processes occurred, in contrast to FEBH (Figure S10). The observed mass loss was related to the evaporation of a small amount of surface-bound water. The TGA further indicated that the first endothermic thermal effect observed in the DSC curves of FEBH-LAC and FEBH-DIA samples in the 70–80 °C temperature range may have come from the presence of some amount of an unreacted coformer. The melting points of pure DIA and LAC were recorded as 77.0 °C and 78 °C, respectively. Moreover, the broad endothermic thermal events observed at 177.5 °C for the FEBH–DIA cocrystal and the endothermic effects observed at 138.3 °C and 176.1 °C were associated with significant mass losses measured in the same temperature range in TGA, suggesting decomposition processes.

## 3. Materials and Methods

### 3.1. Materials

FEBH was purchased from Zhejiang Ausun Pharmaceutical Co., Ltd., Taizhou, China. Analytical reagent-grade materials were used, and they were not purified beforehand. The coformers (purity > 99.8%) were purchased from Sigma-Aldrich, St. Louis, MO, USA. Acetonitrile was obtained from Chempur, Piekary Śląskie, Poland. Water purified from a deionizer-cum-mixed-bed purification system was used in the experiments.

### 3.2. Sample Preparation

Grinding was performed using a Retsch PM100, Verder Scientific Inc, Newtown, PA, USA, with 25 mL stainless steel grinding jars with a 10 mm stainless steel grinding ball at a rate of 30 Hz for 30 min. Experiments were carried out with a 1:1 stoichiometric ratio of FEBH and coformers. Liquid-assisted grinding (LAG) experiments were performed by adding ca. 0.03 mL (1 drop from a pipet) of acetonitrile to the grinding. Grinding without added solvent led to a physical mixture in all cases. The external temperature of the grinding jar after completion of the experiments did not exceed ca. 30 °C. The melting points of new obtained cocrystal were determined on a MPM-H1 capillary melting point apparatus, Schorpp Gerätetechnik, Überlingen, Germany.

### 3.3. Infrared Spectroscopy (FTIR)

FTIR studies were performed using a Perkin-Elmer Spectrum 1000 FT-IR spectrometer, PerkinElmer, Inc., Shelton, Connecticut, USA. All samples were made into a homogenous mixture in KBr using a mortar and a pestle, then gently pressing the powder under vacuum conditions with a compensation force of 10 ton using a 14 mm diameter round flat force punch to produce a KBr pellet. Samples were placed in the light path and the IR spectra from 400 to 4000 cm^−1^ in the transmission mode were obtained.

### 3.4. Powder X-ray Diffraction (PXRD)

Powder diffractograms were recorded on a Bruker D8 Advance (Bruker AXS GmbH, Karlsruhe, Germany) in Bragg–Brentano geometry equipped with a Cu-KR source (λ = 1.54056 Å), 2.5° primary and secondary soller slits, 0.3° divergence slit, an 0.3° antiscatter slit, and a position-sensitive microgap detector, Vantec-1, (Bruker AXS GmbH, Karlsruhe, Germany). The voltage and current applied were 35 kV and 40 mA, respectively. Samples were placed on a sample holder with a 1 mm thickness and 1.5 cm diameter. The data were collected over an angle range of 2 to 50° with a scanning speed of 2° per minute.

### 3.5. Differential Scanning Calorimetry (DSC)

The differential scanning calorimetry measurements were performed on a Mettler-Toledo DSC822e system, Mettler-Toledo Sp. z o.o.,Warsaw, Poland. The samples were prepared in a 40 μL sealed aluminum crucible and heated from 25 °C to 260 °C. The sample mass was about 3–4 mg. Experiments were carried out at a heating rate of 5 °C/min using N_2_ as a protective gas, under flow of 60 mL/min. Data collection and analysis were performed using the program package STARe Software v16.30.

### 3.6. Thermogravimetric Analysis (TGA)

TGA measurements were performed on a Mettler-Toledo TGA/DSC 3+ module, Mettler-Toledo Sp. z o.o.,Warsaw, Poland. The samples were placed in 40 μL aluminum pans and heated from 30 to 260 °C at a rate of 5 °C/min under nitrogen flow of 60 mL/min. The sample mass was about 3–4 mg. The resulting raw TGA curves were corrected for the blank curve. Data collection and analysis were performed using the program package STARe Software v16.30.

### 3.7. Equilibrium Solubility, Dissolution Rate and Stability Studies

The absorption coefficient of each solid phase was measured from the slope of the absorbance-versus-concentration curve for more than five known concentrations of FEB and measured at 314 nm using a UV/visible spectrophotometer (Shimadzu UV-1800), SHIM-POL A.M. Borzymowski, Izabelin, Poland. The solubility of FEB and each obtained solid phase was measured in water after 24 h using the shake-flask method at 37 °C [40]. The experiments were repeated three times.

Solubility studies of FEB and new cocrystals were performed using a Shimadzu UV-1800 spectrometer in distilled water at 37 ± 0.5 °C. The concentrations of FEB and cocrystals were calculated by means of a standard graph, which was made by measuring the absorbance of varied concentrations of FEB at their respective λ_max_. From the slope of the calibration curves, molar extinction coefficients for each cocrystal/FEB were calculated. An excess amount of the sample was added to 5 mL of distilled water medium. The supersaturated solution was agitated using a mechanical shaker at 37 ± 0.5 °C. After 24 h, the suspension was filtered through a Whatman 0.45 mm syringe filter. The filtered aliquots were diluted sufficiently, and the absorbance was measured at λ_max_ = 314 nm for FEBH and its cocrystals.

Dissolution rate experiments were conducted using Erweka DT 80, Verder Scientific Inc, Newtown, PA, USA, with controlled temperature. In this method, distilled water was used as dissolution media. The rate of stirring was 100 ± 2 rpm. In all cases, the amount of the substance tested was 100 mg. The powder was passed through a sieve with a mesh size of 250 µm prior to dissolution testing. The substance was placed in 500 mL of distilled water and maintained at 37 ± 0.5 °C. For 240 min at appropriate intervals (5, 10, 15, 25, 40, 60, 90, 120, 180 and 240 min), 1.5 mL of sample was taken. The dissolution medium was replaced with 1.5 mL of fresh dissolution fluid to maintain a constant volume. The samples were filtered through a 0.45 mm Whatman filter, diluted, and analyzed at 314 nm using a UV/visible spectrophotometer (Shimadzu UV-1800). The average of three determinations, based on a separately constructed calibration curve (R^2^ = 0.999), was used to calculate the dissolution rate of FEBH and the obtained cocrystals. Following the solubility and dissolution rate tests, the undissolved solids were filtered, dried, and subjected to PXRD analysis (Figures S3–S5).

## 4. Conclusions

Our study demonstrates that the stable anhydrous form of FEB cocrystals is consistently obtained when aromatic amides (nicotinamide, isonicotinamide, or picolinamide) are used, regardless of whether FEB is introduced as a pure anhydrous form A or as a hemihydrate. In contrast, obtaining stable FEB cocrystals with aliphatic amides presents significant challenges, as evidenced by the instability of the FEB–acetamide cocrystal.

We found that utilizing FEB hemihydrate (FEBH) for cocrystallization with aliphatic amides—diacetamide (DIA), malonamide (MAL), and D,L-lactamide (LAC)—also led to the formation of anhydrous forms. Additionally, the newly obtained cocrystals exhibited enhanced aqueous solubility compared with FEBH. However, the FEBH–MAL cocrystal was unstable and could not be consistently reproduced.

Among the studied cocrystals, FEBH–LAC exhibited the highest solubility in water (13.9 mg/L); however, it was found that, upon heating, it is prone to undergoing phase transformations. FEB–DIA, on the other hand, demonstrated superior equilibrium solubility (12.2 mg/L) compared with FEBH (7.6 mg/L) and the commercially available A-form (7.5 mg/L), though slightly lower than FEBH–LAC. Importantly, FEBH–DIA displayed the fastest dissolution rate, reaching 6.9 mg/L within five minutes, whereas FEBH–LAC and FEBH reached only 5.8 mg/L and 0.6 mg/L, respectively, in the same time.

These results demonstrate that cocrystallization, particularly with aliphatic amides, is a promising strategy for improving FEB’s solubility and dissolution rate, potentially enhancing its bioavailability and therapeutic effectiveness.

## Figures and Tables

**Table 1:** Melting point of new FEB cocrystals and used coformers.

Drug/Coformer	mp of Coformer (°C)	Cocrystal	mp of Cocrystal (°C)
FEBH	200.5		
DIA	79.0	FEBH-DIA	173.7
MAL	169.8	FEBH-MAL	183.8
LAC	77.0	FEBH-LAC	169.0

**Table 2:** FTIR spectra assignments of major bands of FEBH and the new cocrystals.

Compound	N-H [cm^−1^]	O-H [cm^−1^]	C=N [cm^−1^]	C=O [cm^−1^]	C≡N [cm^−1^]
FEBH		3534 3459 2962	1511	1702 1682	2227
DIA	3272			1747	
	3218 3159 3000			1700	
MAL	3387 3157 2791			1693 1668	
LAC	3389 3303	2986		1651	
FEBH-DIA	3303 3162	2952	1505	1702 1667	2239
FEBH-MAL	3237 3184	2952	1505	1745 1702	2239
FEBH-LAC	3387 3301 3196	2952	1505	1702 1652	2239

## Data Availability

Data are contained within the article and Supplementary Materials.

## Supplementary Materials

The following supporting information can be downloaded at: https://www.mdpi.com/article/10.3390/ijms26073004/s1.
