# Supplementary material for: Enhancing Febuxostat Solubility Through Cocrystal Formation: Role of Substrate Selection and Amide Coformers
Source: Int J Mol Sci. 2025 Mar 26;26(7):3004. doi: 10.3390/ijms26073004 (PMC11988470; doi:10.3390/ijms26073004)
Supplement: Supplementary file 1 [file ijms-26-03004-s001.zip › ijms-3495310-supplementary.pdf]

International Journal of Molecular Sciences

Enhancing Febuxostat Solubility Through Cocrystal Formation: Role of Substrate Selection and Amide Coformers

Edyta Pindelska[1*], Anita Sarna[2], Maciej Duszczyk[2], Anna Zep[3], Izabela D. Madura[4]

[1] Faculty of Pharmacy, Medical University of Warsaw, Banacha 1, 02-093 Warsaw, Poland;

[2] Scientific Circle "Spektrum" at Department of Analytical Chemistry and Biomaterials, Faculty of Pharmacy, Medical University of Warsaw, Banacha 1, 02-093 Warsaw, Poland;

[3] Analytical Research Section, Pharmaceutical Analysis Laboratory, Łukasiewicz Research Network, Industrial Chemistry Institute, Rydygiera 8, 01-793 Warsaw, Poland;

[4] Faculty of Chemistry, Warsaw University of Technology, Noakowskiego 3, 00-664 Warsaw, Poland;

*Corresponding authors: Tel./fax: +48 (22) 57 20 784;

E-mail address: edyta.pindelska@wum.edu.pl;

Contents:

Page 3: Figure S1. PXRD pattern of FEBH (blue), FEB-NIC cocrystal (red), FEB-ISO cocrystal (green), and FEB-PIC cocrystal (dark blue).

Page 4: Figure S2. Comparisons of FTIR spectra of FEBH (blue), DIA (dark green) and FEBH-DIA cocrystal (green).

Page 5: Figure S3. Comparisons of FTIR spectra of FEBH (blue), MAL (violet) and FEBH-MAL cocrystal (magenta).

Page 6: Figure S4. Comparisons of FTIR spectra of FEBH (blue), LAC (dark red) and FEBH-LAC cocrystal (red).

Page 7: Figure S5. Experimental PXRD of FEBH-DIA cocrystal after milling (green), the PXRD obtained after dissolution for 4 h (black) and the prepared physical mixture (yellow). The patterns show the stability of the cocrystal.

Page 7: Figure S6. Experimental PXRD of FEBH-MAL cocrystal after milling (magenta), the PXRD obtained after dissolution for 4 h (black) and the prepared physical mixture (yellow). PXRD pattern of FEBH is shown in blue. The patterns show that the cocrystal is unstable during the dissolution process, and a physical mixture is formed.

Page 8: Figure S7. Experimental PXRD of FEBH-LAC cocrystal after milling (red), the PXRD obtained after dissolution for 4 h (black), the prepared physical mixture (yellow). The patterns show the stability of the cocrystal.

Page 8: Figure S8. Comparisons of DSC curves of FEBH (blue), DIA (black) and FEBH-DIA cocrystal (green).

Page 9: Figure S9. Comparisons of DSC curves of FEBH (blue), LAC (black) and FEBH-LAC cocrystal (red).

Page 9: Figure S10. TGA of FEBH.

Page 10: Figure S11. TGA of FEBH-DIA cocrystal.

Page 10: Figure S12. TGA of FEBH-LAC cocrystal.

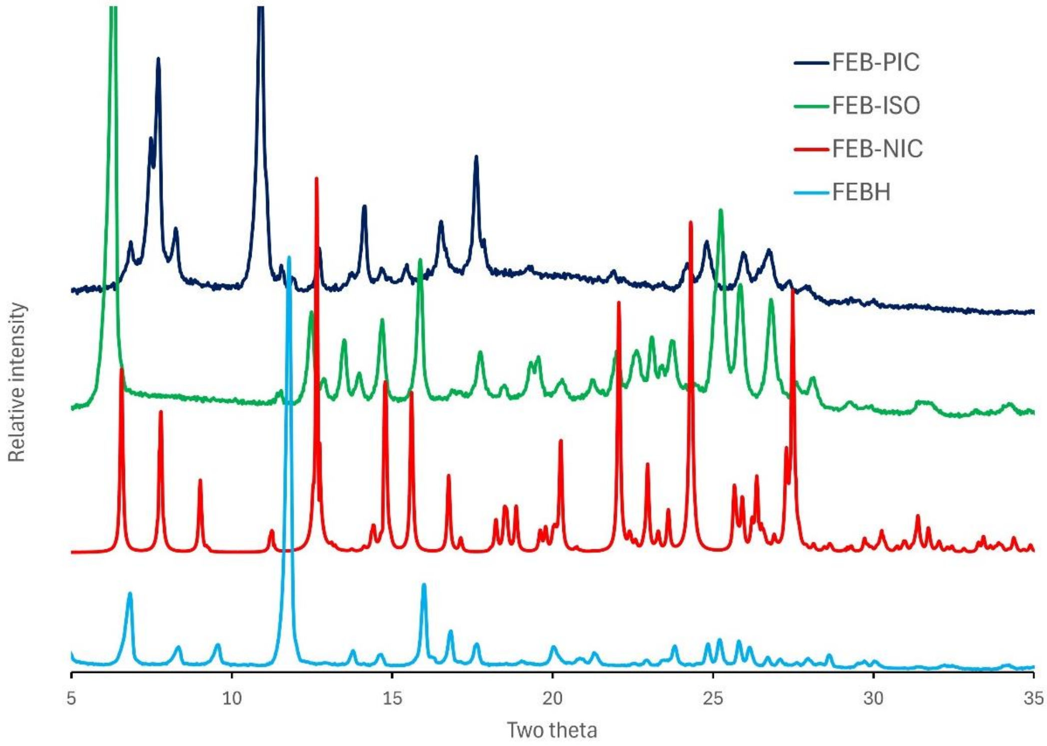

Figure S1. PXRD pattern of FEBH (blue), FEB-NIC cocrystal (red), FEB-ISO cocrystal (green), and FEB-PIC cocrystal (dark blue).

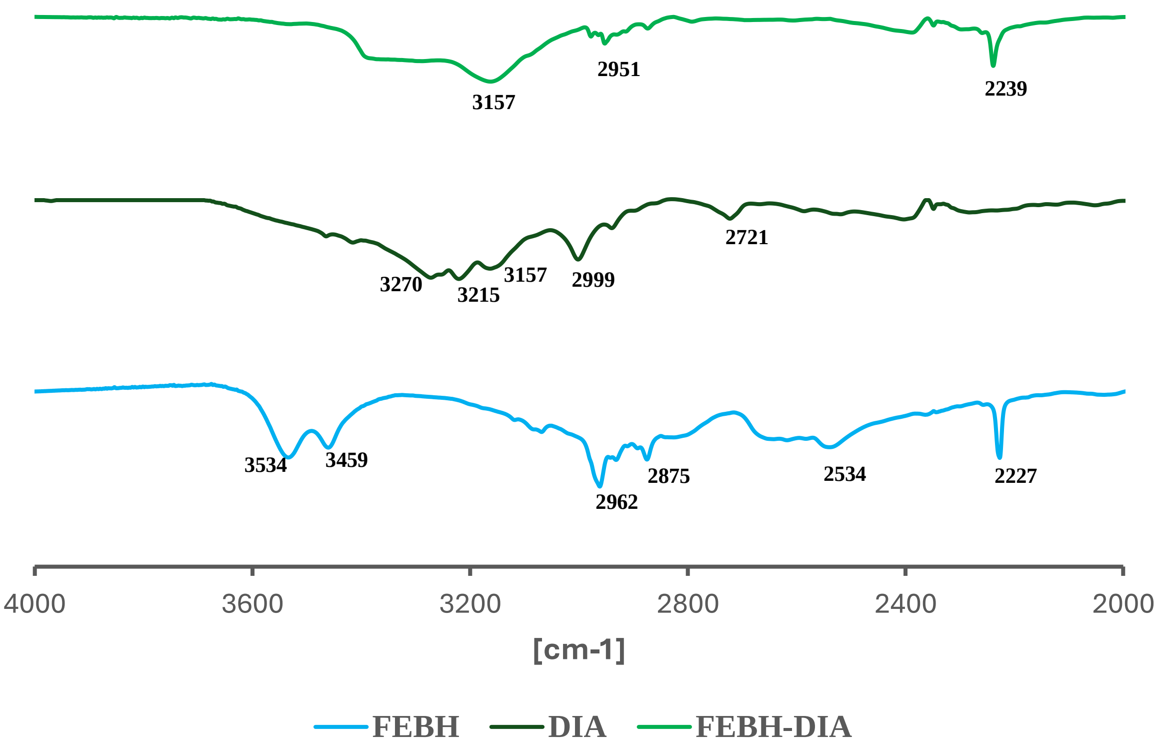

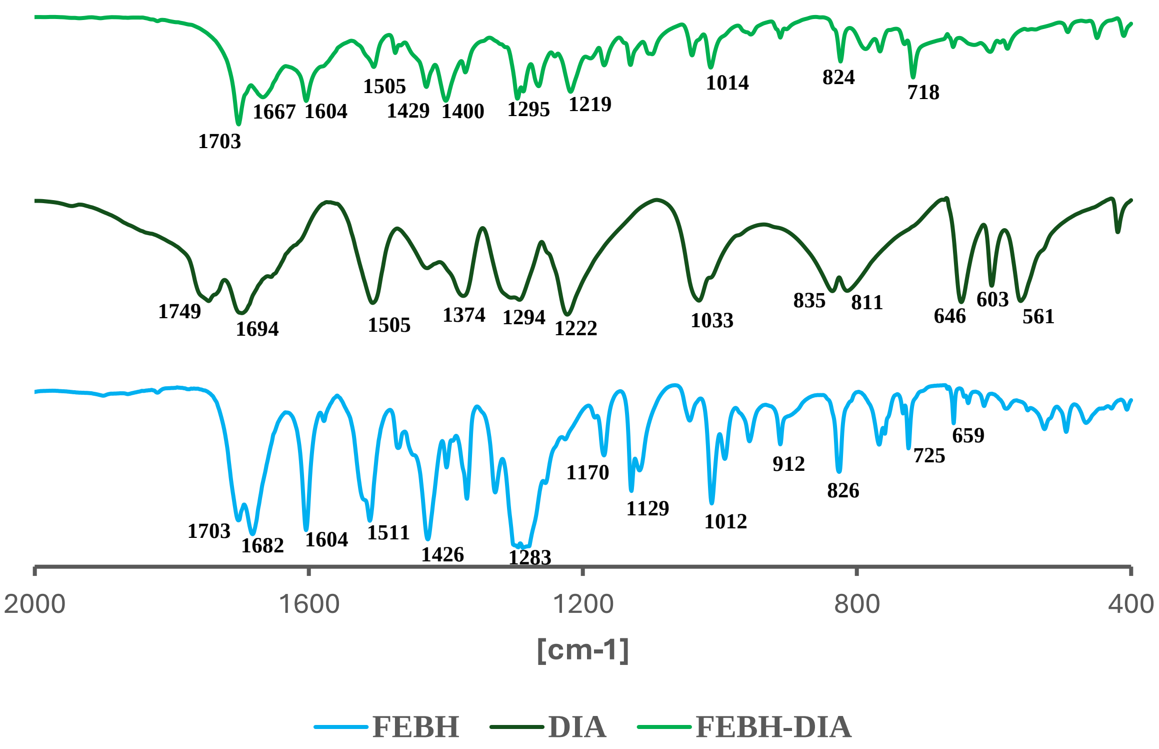

Figure S2. Comparisons of FTIR spectra of FEBH (blue), DIA (dark green) and FEBH-DIA (green).

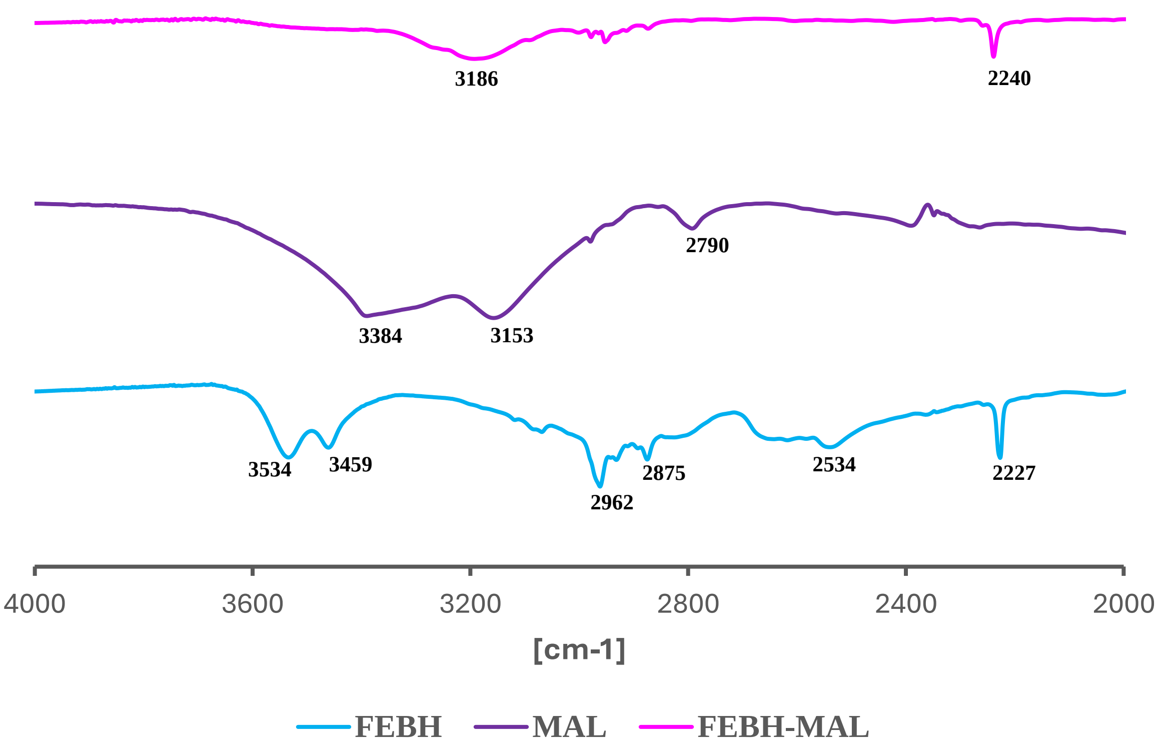

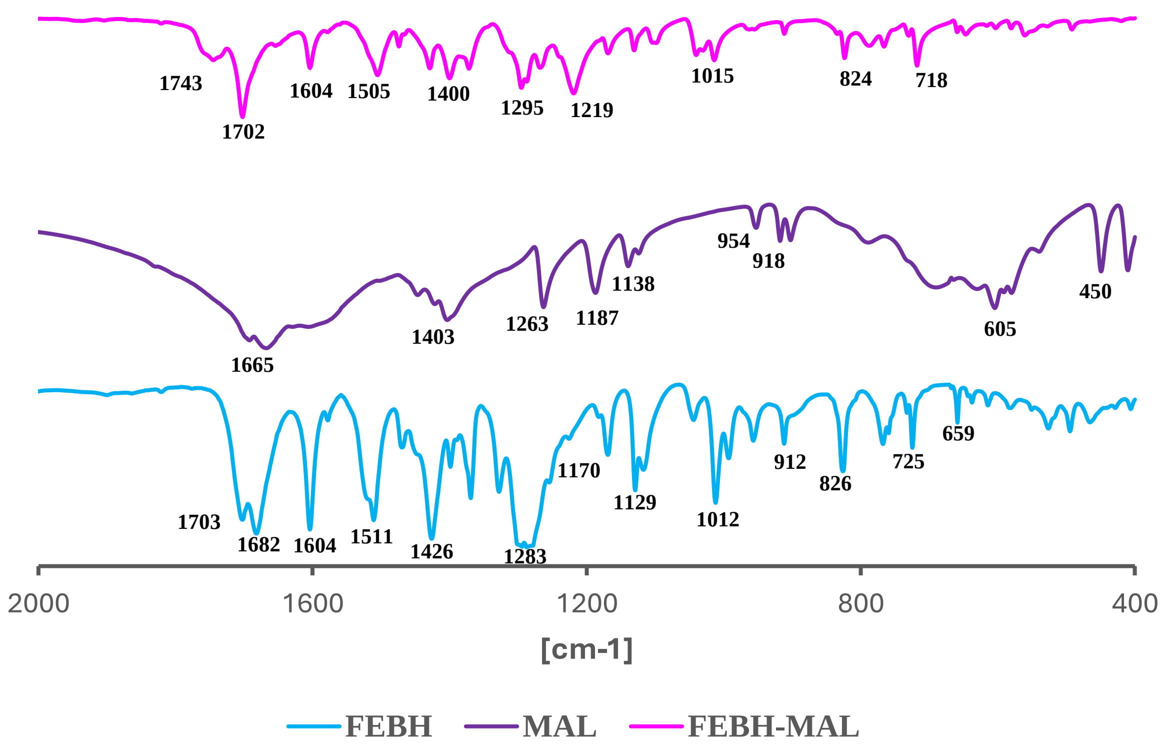

Figure S3. Comparisons of FTIR spectra of FEBH (blue), MAL (violet) and FEBH-MAL (magenta).

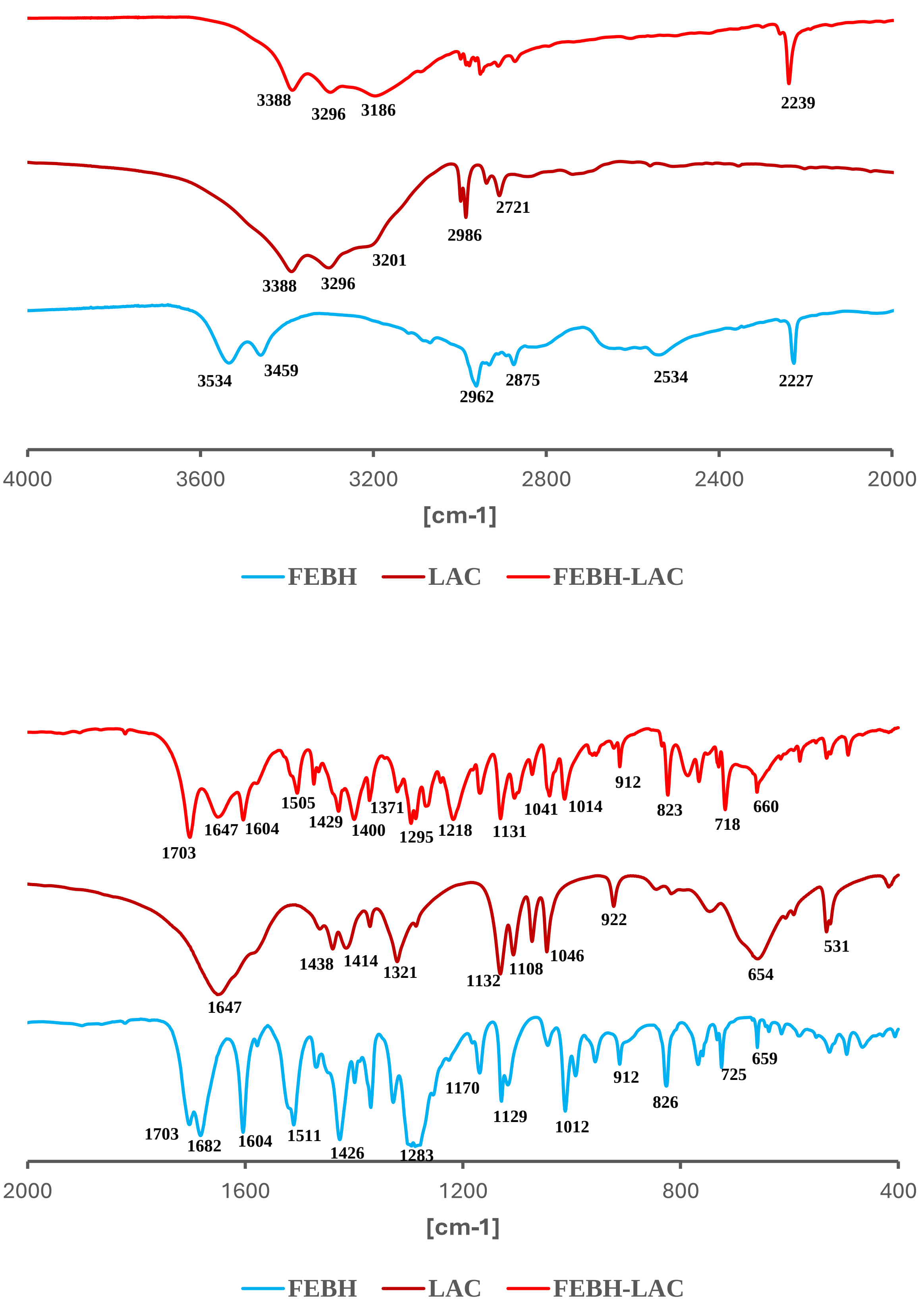

Figure S4. Comparisons of FTIR spectra of FEBH (blue), LAC (dark red) and FEBH-LAC (red).

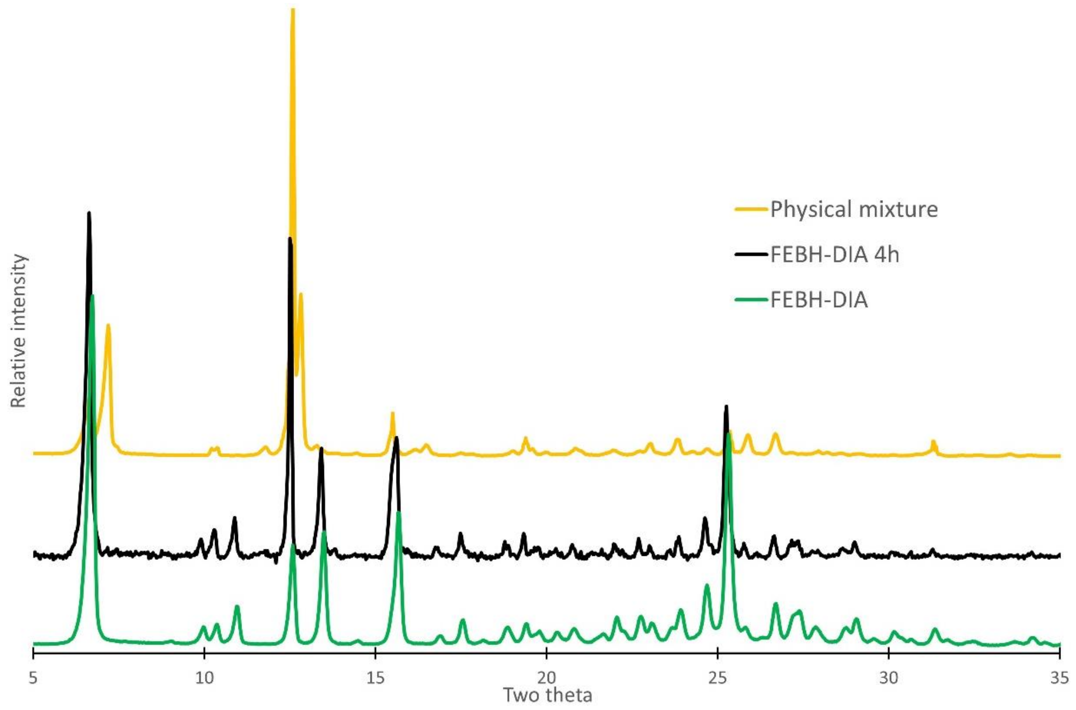

Figure S5. Experimental PXRD of FEBH-DIA cocrystal after milling (green), the PXRD obtained after dissolution for 4 h (black) and the prepared physical mixture (yellow).
The patterns show the stability of the cocrystal.

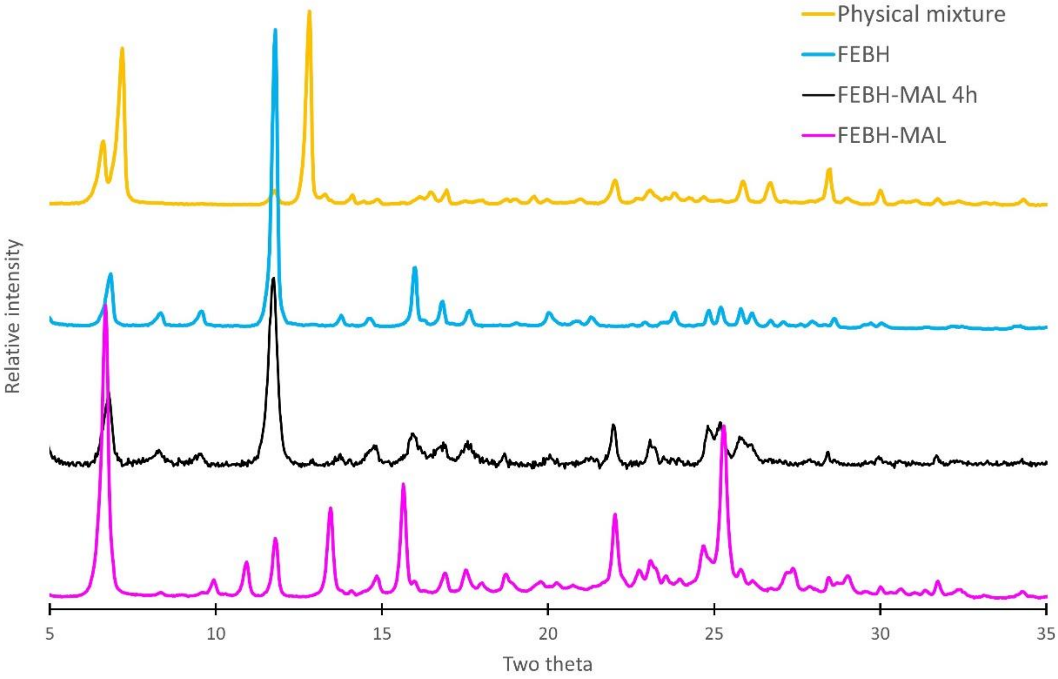

Figure S6. Experimental PXRD of FEBH-MAL cocrystal after milling (magenta), the PXRD obtained after dissolution for 4 h (black) and the prepared physical mixture (yellow). PXRD pattern of FEBH is shown in blue. The patterns show that the cocrystal is unstable during the dissolution process, and a physical mixture is formed.

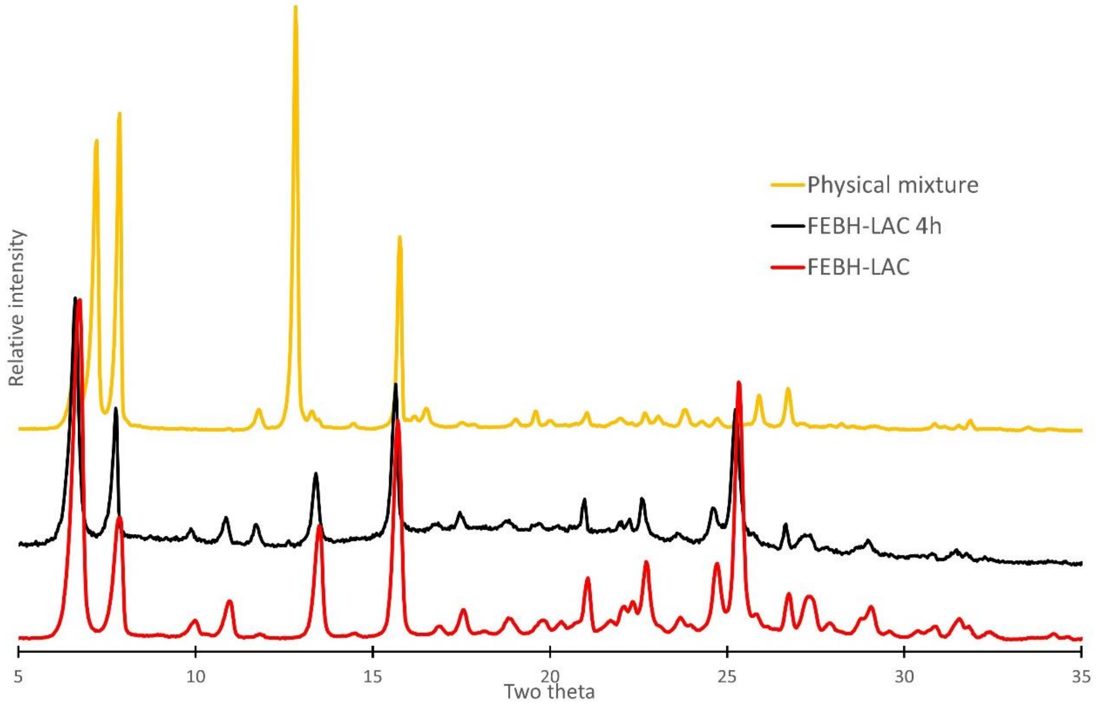

Figure S7. Experimental PXRD of FEBH-LAC cocrystal after milling (red), the PXRD obtained after dissolution for 4 h (black), the prepared physical mixture (yellow).
The patterns show the stability of the cocrystal.

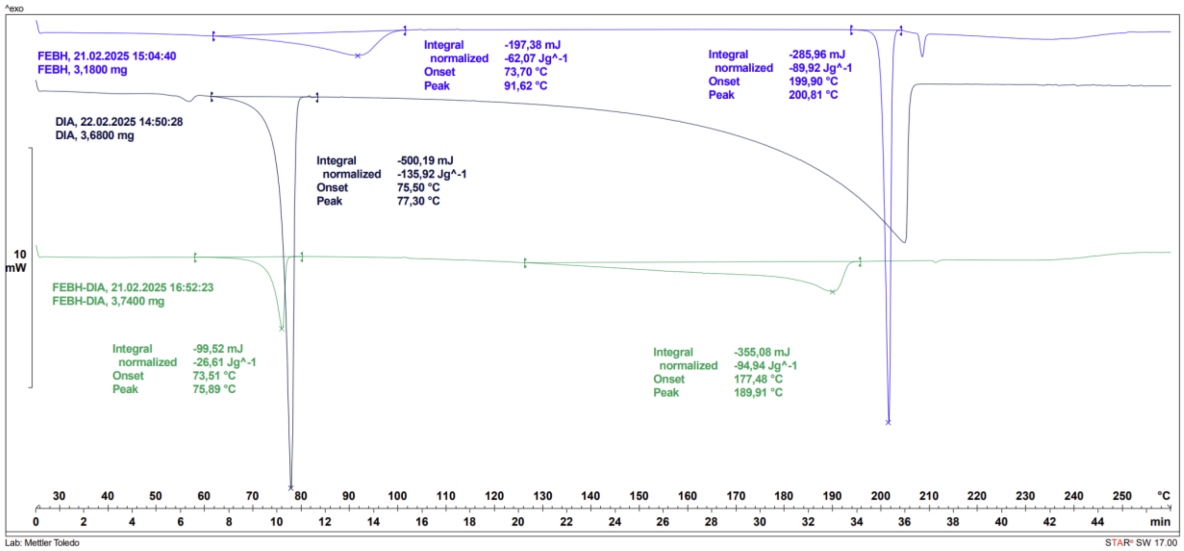

Figure S8. Comparisons of DSC curves of FEBH (blue), DIA (black) and FEBH-DIA cocrystal (green).

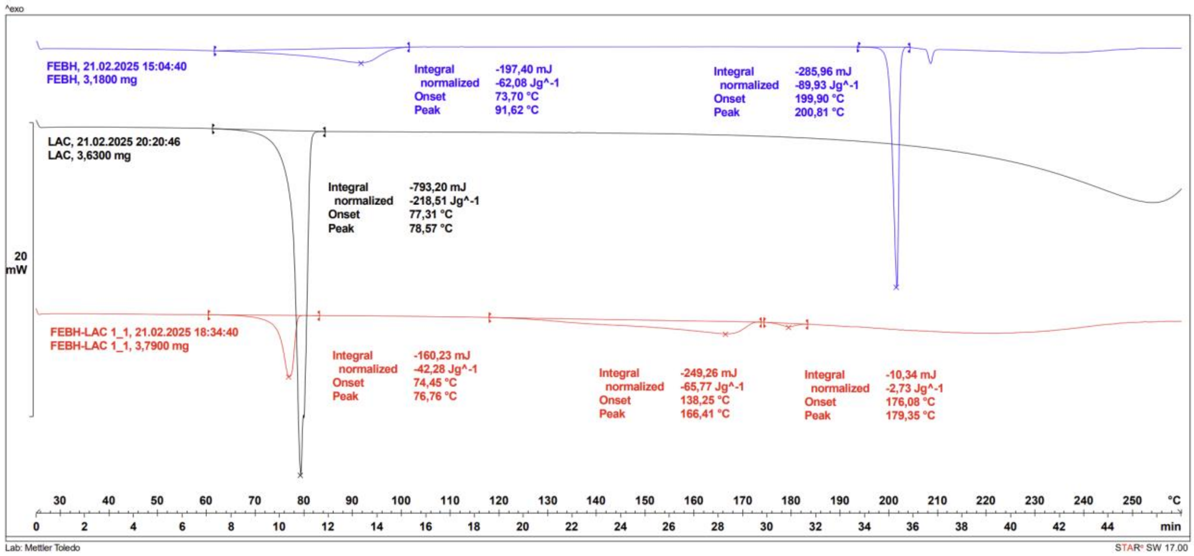

Figure S9. Comparisons of DSC curves of FEBH (blue), LAC (black) and FEBH-LAC cocrystal (red).

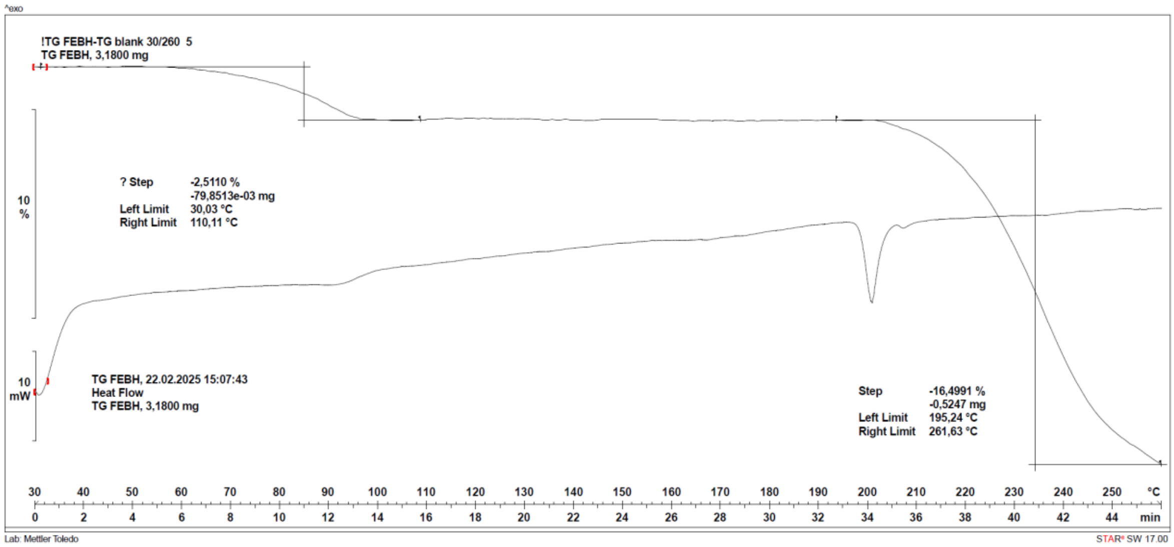

Figure S10. TGA of FEBH.

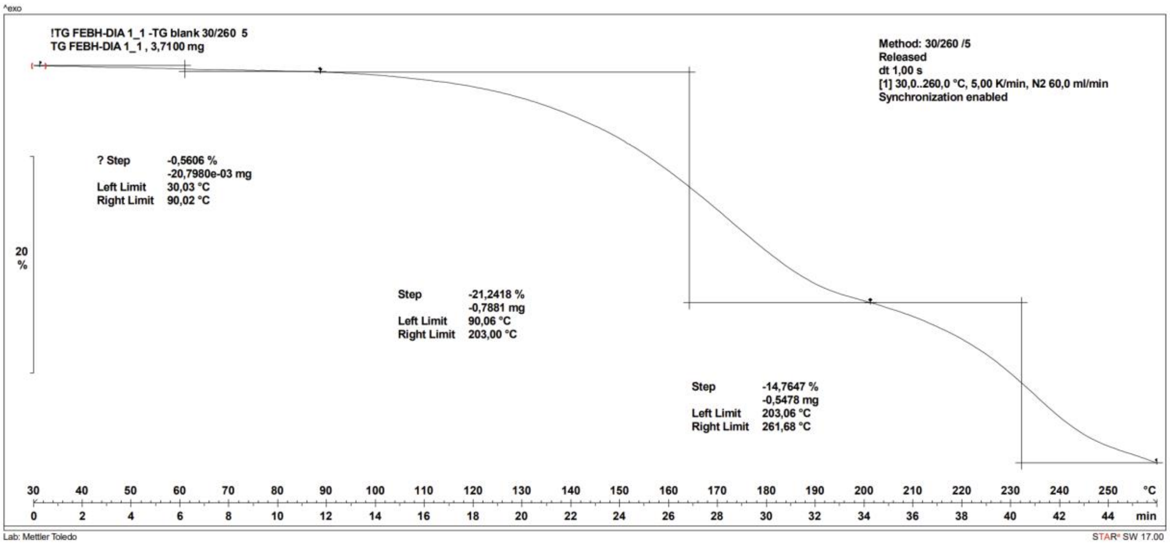

Figure S11. TGA of FEBH-DIA cocrystal.

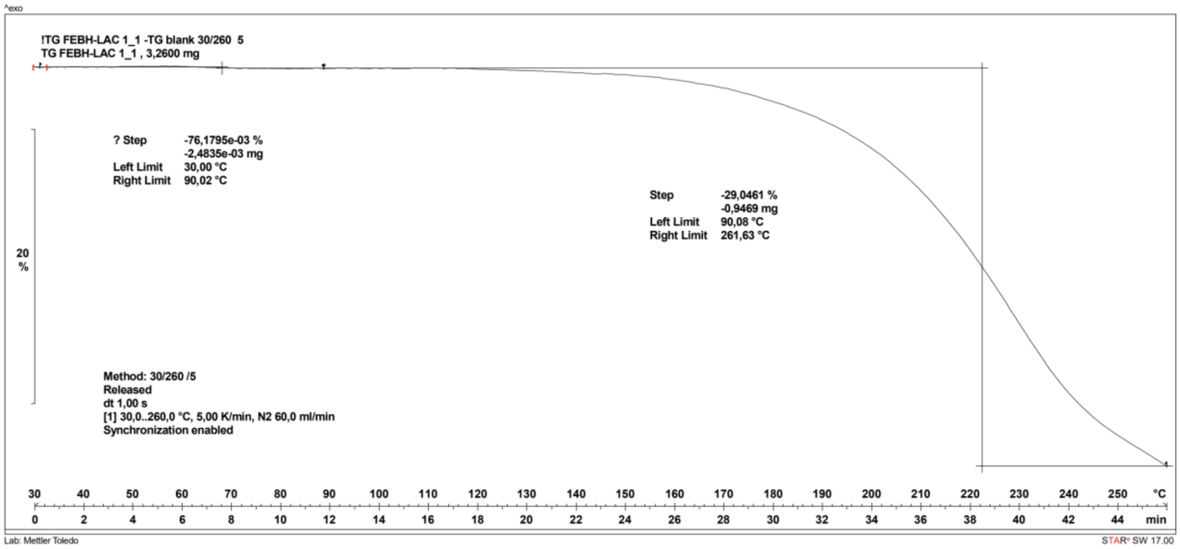

Figure S12. TGA of FEBH-LAC cocrystal.
